# Epacadostat plus pembrolizumab versus placebo plus pembrolizumab as first-line treatment for metastatic non-small cell lung cancer with high levels of programmed death-ligand 1: a randomized, double-blind phase 2 study

**DOI:** 10.1186/s12885-023-11203-8

**Published:** 2024-07-25

**Authors:** Takaaki Tokito, Oleksii Kolesnik, Jens Sørensen, Mehmet Artac, Martín Lázaro Quintela, Jong-Seok Lee, Maen Hussein, Miklos Pless, Luis Paz-Ares, Lance Leopold, Jeannie Daniel, Mihaela Munteanu, Ayman Samkari, Lu Xu, Charles Butts

**Affiliations:** 1 Division of Respirology, Neurology and Rheumatology, Department of Internal Medicine, Kurume University School of Medicine, 67 Asahi-machi, Kurume, Fukuoka 830-0011 Japan; 2 Zaporizhzhya Regional Clinical Oncology Center, Zaporizhzhya State Medical University, Zaporozhye, Ukraine; 3 Department of Oncology, Centre for Cancer and Organ Diseases, Rigshospitalet, Copenhagen, Denmark; 4 Department of Medical Oncology, Necmettin Erbakan Universitesi Meram Tip Fakultesi Hastanesi, Konya, Turkey; 5 Department of Medical Oncology, Hospital Álvaro Cunqueiro-Complexo Hospitalario Universitario de Vigo, Vigo, Spain; 6 Division of Hematology-Oncology, Seoul National University Bundang Hospital, Seongnam, Republic of Korea; 7 Florida Cancer Specialists, Tavares, FL USA; 8 Department of Medical Oncology, Kantonsspital Winterthur, Winterthur, Switzerland; 9 Department of Medical Oncology, Hospital Universitario, 12 de Octubre, Universidad Complutense & Ciberonc, Madrid, Spain; 10 Incyte Corporation, Wilmington, DE United States; 11 Merck & Co., Inc., Rahway, NJ United States; 12 Department of Oncology, Division of Medical Oncology, Cross Cancer Institute, Edmonton, Canada

**Keywords:** Epacadostat, Combination immunotherapy, Non-small cell lung cancer, Pembrolizumab, PD-L1 high

## Abstract

**Background:**

Pembrolizumab is a first-line therapy for certain patients with advanced/metastatic non-small cell lung cancer (NSCLC). Combining pembrolizumab with other immunotherapies may enhance tumor cell killing and clinical outcomes. Epacadostat is a selective inhibitor of indoleamine 2,3-dioxygenase 1, an immuno-regulatory enzyme involved in tryptophan to kynurenine metabolism that inhibits T cell-mediated immune responses.

**Methods:**

In this randomized phase II study, patients with metastatic NSCLC expressing high (≥ 50%) programmed death-ligand 1 (PD-L1) levels received pembrolizumab 200 mg every 21 days plus oral epacadostat 100 mg twice daily (combination) or matching placebo (control). The primary objective was objective response rate (ORR); secondary objectives were progression-free survival (PFS), overall survival (OS), duration of response (DOR) and safety/tolerability.

**Results:**

154 patients were randomized (77 per group). Median (range) follow-up was 6.8 months (0.1–11.4) and 7.0 months (0.2–11.9) in the combination and control groups, respectively Confirmed ORR was similar between groups (combination: 32.5%, 95% CI 22.2–44.1; control: 39.0%, 95% CI 28.0–50.8; difference: − 6.5, 95% CI − 21.5 to 8.7; 1-sided *P* = 0.8000). Median (range) DOR was 6.2 months (1.9 + to 6.5 +) and not reached (1.9 + to 8.6 +) in the combination and control groups, respectively. Although not formally tested, median PFS was 6.7 and 6.2 months for the combination and control groups, respectively, and median OS was not reached in either group. Circulating kynurenine levels increased from C1D1 to C2D1 (*P* < 0.01) in the control group and decreased from C1D1 to C2D1 (*P* < 0.01) in the combination group but were not normalized in most patients. The most frequent serious adverse events (AEs) (≥ 2%) were pneumonia (4.0%), anemia (2.7%), atelectasis (2.7%) and pneumonitis (2.7%) in the combination group and pneumonia (3.9%), pneumonitis (2.6%) and hypotension (2.6%) in the control group. Two deaths due to drug-related AEs were reported, both in the control group.

**Conclusions:**

Addition of epacadostat to pembrolizumab therapy for PD-L1–high metastatic NSCLC was generally well tolerated but did not demonstrate an improved therapeutic effect. Evaluating higher doses of epacadostat that normalize kynurenine levels when given in combination with checkpoint inhibitors may be warranted.

**Trial registration:**

ClinicalTrials.gov, NCT03322540. Registered 10/26/2017.

**Supplementary Information:**

The online version contains supplementary material available at 10.1186/s12885-023-11203-8.

## Background

Approximately 84% of all lung cancers are non-small cell lung cancers (NSCLC) [1]. At diagnosis, most patients with NSCLC have advanced disease, which is generally not curable [1, 2]. Pembrolizumab monotherapy is the current first-line standard-of-care therapy for patients with advanced or metastatic NSCLC tumors expressing programmed death-ligand 1 (PD-L1) and with no EGFR or ALK genomic tumor aberrations [3–6]. However, there are multiple ways cancer cells escape the host immune response [7]. Combining pembrolizumab with other immunotherapy approaches may provide enhanced immune-mediated killing of tumor cells and further increase therapeutic benefit.

Indoleamine 2,3-dioxygenase 1 (IDO1) is an immuno-regulatory enzyme involved in the metabolism of tryptophan to kynurenine [8]. Upregulated expression of the IDO1 enzyme is associated with dampened anticancer T-cell immunity [8, 9]). Many human tumor types constitutively express IDO1 [8, 10]. Co-expression of IDO1 and PD-L1 is found in some NSCLC tumors, but different studies have reported varying degrees of co-expression [11–13]. Furthermore, regardless of baseline expression levels, IDO1 can counter anticancer inflammatory immune responses because it is induced by interferon-y [8, 14–17]. Thus, IDO1 inhibition may facilitate the activity of checkpoint inhibitors by preventing this resistance mechanism. Epacadostat is a potent selective oral inhibitor of IDO1 [18, 19]; twice-daily (BID) epacadostat monotherapy at doses ≥ 100 mg in patients with advanced solid tumors reduced plasma kynurenine to levels observed in healthy volunteers [19].

A number of clinical trials were initiated to investigate the potential of combining epacadostat and pembrolizumab to improve outcomes in several cancers, including NSCLC. Promising efficacy was observed in the melanoma and a NSCLC cohort of the phase I/II ECHO-202/KEYNOTE-037 study [20] assessing epacadostat plus pembrolizumab for advanced tumors. For patients with previously treated NSCLC, the objective response rate (ORR) was 24.4% in the PD-L1 tumor proportion score (TPS) < 50% group. In a small number of patients with NSCLC and PD-L1 TPS ≥ 50%, the ORR was 30.8% [21]. Here, we present results from the primary analysis of a randomized phase II study assessing the safety and efficacy of epacadostat plus pembrolizumab (combination) versus placebo plus pembrolizumab (control) in patients with metastatic NSCLC expressing high levels of PD-L1 (TPS ≥ 50%) (NCT03322540).

## Methods

### Study design and conduct

ECHO-305/KEYNOTE-654 was a multicenter, active-controlled, double-blind, parallel-group randomized phase II study. This study was originally designed as a phase III study. On May 31, 2018, the protocol was amended to a phase II study after emerging data from the phase III ECHO-301/KEYNOTE-252 study in unresectable or metastatic melanoma showed that addition of epacadostat to pembrolizumab did not improve the primary endpoint of progression-free survival (PFS) [22]. Specific changes made in this protocol amendment are detailed in the relevant methods sections below.

This study conformed to the ethical principles of the Declaration of Helsinki, Good Clinical Practice, and applicable country and/or local statutes and regulations.

### Study population

Patients ≥ 18 years old with previously untreated, confirmed stage IV NSCLC not suitable for primary EGFR-, ALK- or ROS1-directed therapy and measurable disease per Response Evaluation Criteria in Solid Tumors version 1.1 (RECIST v1.1), an Eastern Cooperative Oncology Group performance status (ECOG PS) of 0 or 1, and tumor tissue with PD-L1 TPS ≥ 50% were eligible. Exclusion criteria included any prior treatment for metastatic NSCLC and untreated central nervous system metastases and/or carcinomatous meningitis.

### Study procedure and interventions

Patients were randomized to receive treatment in one of two arms: epacadostat plus pembrolizumab (combination) or placebo plus pembrolizumab (control). The stratification factors in the original study design were tumor histology (squamous vs. nonsquamous), ECOG PS and geographical region. The protocol amendment changing the study to a phase II study updated the study design so that tumor histology (squamous vs. nonsquamous) was the only stratification factor.

Pembrolizumab 200 mg was administered intravenously every 21 days (day 1 of each cycle) for up to 35 doses and epacadostat 100 mg or matching placebo was administered orally BID. Pembrolizumab could be withheld for up to 12 weeks from the last dose to mitigate immune-related adverse events (AEs). Epacadostat could be reduced to 50 or 25 mg BID to mitigate immune-related AEs. Discontinuation of study therapy due to disease progression was based on immune-related RECIST criteria (iRECIST) as evaluated by investigators. Blood was drawn from fasted patients before dosing, on day 1 of cycle 1 (C1D1) and day 1 of cycle 2 (C2D1). Serum kynurenine levels were determined by a proprietary validated liquid chromatography–tandem mass spectrometry assay using calibrated standards at Worldwide Clinical Trials, Morrisville, NC.

### Study objectives and endpoints

In the original study design, the primary endpoints were overall survival (OS) and PFS. The protocol amendment changed the study to a phase II study with the primary objective comparing ORR of the combination and control groups. Response and disease progression were assessed by blinded independent central review (BICR) based on modified RECIST v1.1 criteria allowing a maximum of 10 target lesions in total and five per organ. The secondary objectives were PFS, OS, duration of response (DOR) and safety/tolerability. National Cancer Institute Common Terminology Criteria for Adverse Events (NCI CTCAE) version 4.0 was used to grade and record AEs. The pharmacodynamic activity of epacadostat, assessed by changes in circulating kynurenine levels from baseline, was among the exploratory objectives.

### Statistical analyses

Originally, 588 patients were planned for enrollment in the phase III study. Target enrollment was reduced to 148 patients when the study was amended to a phase II study. The efficacy analysis included all randomized patients (i.e., the intention-to-treat population), and the safety analysis included all patients who received at least one treatment dose. ORR was compared between treatment arms using the Miettinen and Nurminen method [23] stratified by predominant tumor histology (squamous vs. non-squamous). Based on the number of patients planned to be randomized (*N* = 148), the study had 81.7% power to detect a 20-percentage point difference in ORR between combination and control groups at α = 5% (one-sided). PFS and OS were compared between treatment arms using a stratified log-rank test. Event rates were estimated using the Kaplan–Meier method, and hazard ratios (HRs) were estimated using a stratified Cox regression model with Efron’s method of tie handling. Circulating kynurenine levels were compared within each treatment arm using paired t-tests.

## Results

### Patient characteristics

**Fig. 1:**
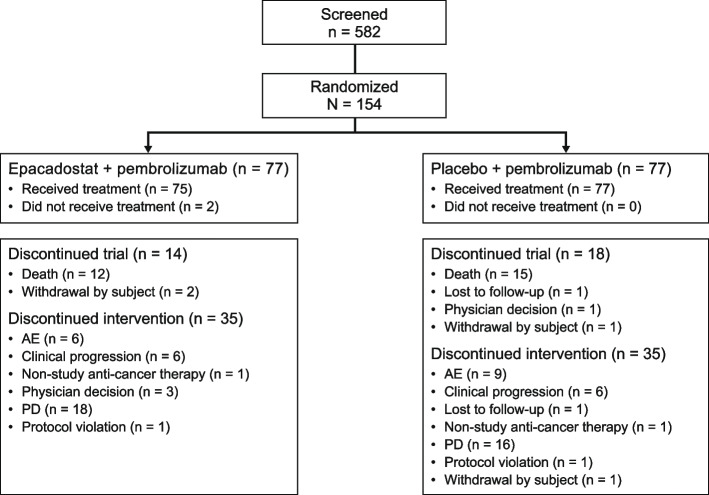
Patient disposition. *AE* adverse event, *PD* progressive disease

A total of 154 patients were randomized (1:1) to combination (*n* = 77) or control (*n* = 77) treatment arms (Fig. 1). Most patients remained in the study at data cutoff. The majority were male, white, non-Hispanic or Latino, older than 65 years of age, former smokers, with an ECOG PS of 1 and a metastatic stage of M1c (with a slightly higher occurrence in the combination group) (Table 1). More patients were older than 65 years in the control group compared with the combination group. The predominant tumor histology was balanced between the combination and control groups.

**Table 1:** Patient and disease characteristics

	Epacadostat + pembrolizumab (*n* = 77)	Placebo + pembrolizumab (*n* = 77)
Gender		
Male	53 (68.8)	59 (76.6)
Female	24 (31.2)	18 (23.4)
Age, years		
< 65	40 (51.9)	31 (40.3)
≥ 65	37 (48.1)	46 (59.7)
Median (range)	64.0 (37–89)	69.0 (40–85)
Race		
White	52 (67.5)	54 (70.1)
Asian	25 (32.5)	23 (29.9)
Ethnicity		
Not Hispanic or Latino	66 (85.7)	73 (94.8)
Hispanic or Latino	7 (9.1)	4 (5.2)
Not reported	3 (3.9)	0
Unknown	1 (1.3)	0
Smoking status		
Never	8 (10.4)	9 (11.7)
Former	51 (66.2)	54 (70.1)
Current	18 (23.4)	14 (18.2)
ECOG PS		
0	23 (29.9)	20 (26.0)
1	54 (70.1)	57 (74.0)
Predominant tumor histology		
Squamous	20 (26.0)	22 (28.6)
Nonsquamous	57 (74.0)	55 (71.4)
Metastatic stage		
M0	1 (1.3)	0
M1a	19 (24.7)	22 (28.6)
M1b	17 (22.1)	22 (28.6)
M1c	40 (51.9)	33 (42.9)
Brain metastasis status at baseline		
Yes	7 (9.1)	6 (7.8)
No	70 (90.9)	71 (92.2)
Prior adjuvant therapy		
Yes	5 (6.5)	2 (2.6)
Prior radiation		
Yes	16 (20.8)	14 (18.2)
No	61 (79.2)	63 (81.8)

*ECOG PS* Eastern Cooperative Oncology Group performance status

Data are *n* (%) unless otherwise noted

No patients had prior neoadjuvant therapy

### Treatment duration

The median number of days on treatment, days on pembrolizumab, and days on epacadostat/placebo were all similar for both treatment groups (see Additional file 1). The median follow-up was 6.8 months (range 0.1–11.4) in the combination group and 7.0 months (range 0.2–11.9) in the control group. Upon study termination, treatments were unblinded and epacadostat was discontinued. All remaining patients had the option to continue open-label pembrolizumab monotherapy.

### Efficacy

The confirmed ORR based on BICR was similar in both treatment groups at 32.5% (95% confidence interval [CI] 22.2–44.1) in the combination group compared with 39.0% (95% CI 28.0–50.8) in the control group (Table 2). The difference in estimated ORR percentage between groups was − 6.5 (95% CI − 21.5 to 8.7; one-sided *P* = 0.8000). More patients in the control group (39.0%) had a best overall response of partial response compared with the combination group (32.5%), while more patients in the combination group had a best overall response of stable disease (41.6%) compared with the control group (29.9%). The disease control rate was numerically higher in the combination group compared with the control group. The median DOR in the combination and control group was 6.2 months (range 1.9 + to 6.5 +) and not reached (range 1.9 + to 8.6 +), respectively (plus symbols indicate no progressive disease at the time of last disease assessment) (Table 2).

**Table 2:** Summary of objective response

	Epacadostat + pembrolizumab (*n* = 77)		Placebo + pembrolizumab (*n* = 77)	
	*n*	% (95% CI)	*n*	% (95% CI)
CR	0	0.0 (0.0–4.7)	0	0.0 (0.0–4.7)
PR	25	32.5 (22.2–44.1)	30	39.0 (28.0–50.8)
Overall response^a^	25	32.5 (22.2–44.1)	30	39.0 (28.0–50.8)
SD^b^	32	41.6 (30.4–53.4)	23	29.9 (20.0–41.4)
Disease control^c^	57	74.0 (62.8–83.4)	53	68.8 (57.3–78.9)
PD	12	15.6 (8.3–25.6)	15	19.5 (11.3–30.1)
NE^d^	1	1.3 (0.0–7.0)	2	2.6 (0.3–9.1)
No assessment^e^	7	9.1 (3.7–17.8)	7	9.1 (3.7–17.8)
Patients with a response^f^	*n* = 25		*n* = 30	
Median TTR, months (range)	2.1 (1.1–4.2)		2.1 (1.1–4.2)	
Median DOR,^g^ months (range)	6.2 (1.9 + to 6.5 +)		NR (1.9 + to 8.6 +)	
Patients with an ongoing response, *n* (%)	21 (84.0)		27 (90.0)	
≥ 6 months	1 (4.0)		13 (43.3)	

*BICR* blinded independent central review, *CI* confidence interval, *CR* complete response, *DOR* duration of response, *NE* not evaluable, *NR* not reached, *PD* progressive disease, *PR* partial response, *RECIST v1.1* Response Evaluation Criteria in Solid Tumors version 1.1, *SD* stable disease, *TTR* time to response

Responses based on BICR assessment per RECIST v1.1

^a^Overall response includes CR and PR

^b^SD includes both SD and Non-CR/Non-PD

^c^Disease control includes CR, PR and SD

^d^Postbaseline assessment(s) available but not evaluable or CR/PR/SD < 6 weeks from randomization

^e^No postbaseline assessment available

^f^Includes patients with best objective response as confirmed CR or PR

^g^From product-limit (Kaplan–Meier) method for censored data. " + " indicates no PD was reported by the time of last disease assessment

**Fig. 2:**
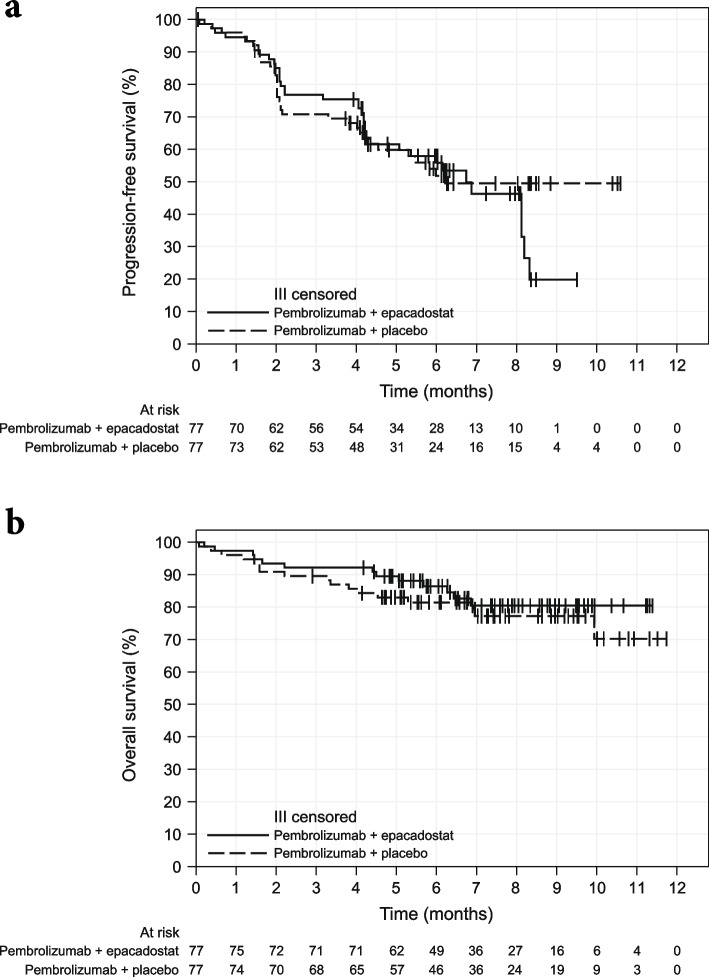
Kaplan–Meier curves for **a** PFS^a^ and **b** OS. ^a^Based on BICR assessment per RECIST v1.1. *BICR* blinded independent central review, *OS* overall survival, *PFS* progression-free survival, *RECIST v1.1* Response Evaluation Criteria in Solid Tumors version 1.1

At data cutoff, PFS data were not conclusive, with 71 of the 95 required PFS events having been reported (37/77 [48.1%] in the combination group; 34/77 [44.2%] in the control group) (Fig. 2A, Table 3). Median PFS was 6.7 months for the combination group and 6.2 months for the control group (HR 1.10, 95% CI 0.69–1.76). The PFS rates at 3 and 6 months were similar for both groups. The median OS was not reached in either group (combination: 13 events; control: 17 events; HR 0.74, 95% CI 0.36–1.52) (Fig. 2B, Table 3).

**Table 3:** Analysis of PFS and OS

	**PFS**		**OS**	
	Epacadostat + pembrolizumab (*n* = 77)	Placebo + pembrolizumab (*n* = 77)	Epacadostat + pembrolizumab (*n* = 77)	Placebo + pembrolizumab (*n* = 77)
Number of events, n (%)	37 (48.1)	34 (44.2)	13 (16.9)	17 (22.1)
Median^a^ (95% CI), months	6.7 (4.3–8.2)	6.2 (4.3–NE)	NE (NE–NE)	NE (NE–NE)
Rate at month 6 in %^a^ (95% CI)	58.0 (45.2–68.8)	51.9 (38.9–63.4)	86.4 (76.2–92.5)	81.5 (70.7–88.6)
Hazard ratio (95% CI)^b^	1.10 (0.69–1.76)		0.74 (0.36–1.52)	
*P* value	0.659^c^		0.205^c^	

*BICR* blinded independent central review, *CI* confidence interval, *NE* not evaluated, *OS* overall survival, *PFS* progression-free survival, *RECIST v1*.*1* Response Evaluation Criteria in Solid Tumors version 1.1

Disease progression based on BICR assessment per RECIST v1.1

^a^From product-limit (Kaplan–Meier) method for censored data

^b^Based on Cox regression model with Efron’s method of tie handling with treatment as a covariate stratified by tumor histology (squamous vs. nonsquamous)

^c^One-sided *P* value based on log-rank test stratified by tumor histology (squamous vs. nonsquamous)

Subgroup analyses showed results similar to those from the overall analyses (ORR, PFS and OS) (data on file). Subgroups included predominant tumor histology, age, gender, race (white vs. nonwhite), smoking status, geographic region (East Asian vs. non-East Asian), baseline ECOG status, baseline metastatic stage and history of brain metastasis. Investigator assessments were consistent with BICR assessments for ORR, PFS and DOR.

### Safety and tolerability

The proportions of patients with AEs, drug-related AEs, grade ≥ 3 AEs, drug-related grade ≥ 3 AEs, serious AEs (SAEs) and drug-related SAEs were similar between treatment groups (Table 4). The most frequent SAEs (≥ 2%) in the combination group were pneumonia (4.0%), anemia (2.7%), atelectasis (2.7%) and pneumonitis (2.7%) and in the control group pneumonia (3.9%), pneumonitis (2.6%) and hypotension (2.6%). All drug-related SAEs were reported by ≤ 2 patients. Two deaths due to drug-related AEs were reported in the control group, one from pneumonia and the other from respiratory failure. No deaths due to drug-related AEs were reported in the combination group.

**Table 4:** Summary of adverse events

	Epacadostat + pembrolizumab (*n* = 75)		Placebo + pembrolizumab (*n* = 77)	
	Any AE, n (%)	Drug-related AE, *n* (%)	Any AE, n (%)	Drug-related AE, *n* (%)
Any AE^a^	69 (92.0)	53 (70.7)^b^	72 (93.5)	49 (63.6)^b^
Constipation	18 (24.0)	4 (5.3)	16 (20.8)	4 (5.2)
Decreased appetite	14 (18.7)	9 (12.0)	8 (10.4)	3 (3.9)
Diarrhea	13 (17.3)	7 (9.3)	15 (19.5)	10 (13.0)
Nausea	12 (16.0)	8 (10.7)	7 (9.1)	3 (3.9)
Pruritus	9 (12.0)	8 (10.7)	12 (15.6)	11 (4.3)
Fatigue	7 (9.3)	5 (6.7)	14 (18.2)	8 (10.4)
Grade ≥ 3 AE	37 (49.3)	16 (21.3)	34 (44.2)	19 (24.7)
SAE	25 (33.3)	7 (9.3)	26 (33.8)	10 (13.0)
Discontinued due to an AE	10 (13.3)	7 (9.3)	9 (11.7)	6 (7.8)
Pembrolizumab	5 (6.7)	3 (4.0)	8 (10.4)	5 (6.5)
Epacadostat/placebo	10 (13.3)	7 (9.3)	9 (11.7)	6 (7.8)
Epacadostat/placebo and pembrolizumab	5 (6.7)	3 (4.0)	8 (10.4)	5 (6.5)
Discontinued due to an SAE	5 (6.7)	2 (2.7)	6 (7.8)	3 (3.9)
Pembrolizumab	4 (5.3)	2 (2.7)	6 (7.8)	3 (3.9)
Epacadostat/placebo	5 (6.7)	2 (2.7)	6 (7.8)	3 (3.9)
Epacadostat/placebo and pembrolizumab	4 (5.3)	2 (2.7)	6 (7.8)	3 (3.9)
Deaths	3 (4.0)	0	7 (9.1)	2 (2.6)

*AE* adverse event, *CTCAE* Common Terminology Criteria for Adverse Events, *MedDRA* Medical Dictionary for Regulatory Activities, *NCI* National Cancer Institute, *SAE* serious adverse event

Nonserious AEs up to 30 days of last dose and serious AEs up to 90 days of last dose are included

Grades are based on NCI CTCAE version 4.0

MedDRA preferred terms "Neoplasm progression", "Malignant neoplasm progression" and "Disease progression" not related to the drug are excluded

^a^AEs (any grade) in ≥ 15% of patients in the combination or control treatment arms

^b^Determined by the investigator to be related to the drug

### Pharmacodynamic activity of epacadostat

**Fig. 3:**
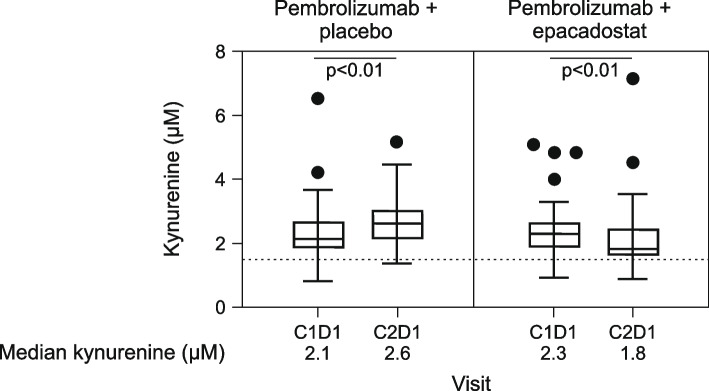
Circulating kynurenine levels at baseline (C1D1) and after one cycle of treatment (C2D1). The number of samples assessed was 56 in the pembrolizumab plus placebo group and 57 in the pembrolizumab plus epacadostat group. Kynurenine levels at C1D1 and C2D1 were compared using paired t-tests within each treatment arm. The dotted line indicates median kynurenine levels in healthy subjects (1.5 μM) [19]. *C* cycle, *D* day

Median baseline levels of circulating kynurenine in both treatment arms (Fig. 3) were numerically above that observed in healthy subjects (1.5 μM) [19]. Compared with baseline levels (C1D1), median circulating kynurenine levels were reduced after one cycle of treatment (C2D1) in the combination group (2.3 µM vs. 1.8 µM; *P* < 0.01), albeit not to levels reported in healthy volunteers. The opposite was observed for the control group where compared with C1D1, median circulating kynurenine levels were increased at C2D1 (2.1 µM vs. 2.6 µM; *P* < 0.01).

## Discussion

In the ECHO-305/KEYNOTE-654 study, the addition of epacadostat 100 mg BID to pembrolizumab did not improve ORR in patients with previously untreated metastatic PD-L1 TPS ≥ 50% NSCLC. The PFS data were not conclusive to confirm an effect of combination therapy with epacadostat plus pembrolizumab. Although baseline characteristics were generally balanced between treatment arms, a higher proportion of M1C patients in the combination arm may have affected ORR comparisons between arms. The combination of epacadostat plus pembrolizumab was generally well tolerated with an acceptable safety profile that was generally consistent with that of pembrolizumab monotherapy with respect to AEs and treatment discontinuations due to AEs. No new safety concerns were identified.

This study was based on the phase III KEYNOTE-024 [5] and the phase I/II ECHO-202/KEYNOTE-037 [20] studies. The ORR of the epacadostat plus pembrolizumab group in the current study (32.5%) was similar to that in the open-label PD-L1 TPS ≥ 50% group in ECHO-202/KEYNOTE-037 (30.8%) [21]. Also, the ORR of the placebo plus pembrolizumab group in this study (39.0%) was similar to that in the pembrolizumab monotherapy group of the phase III KEYNOTE-024 study (44.8%), which also included patients with previously untreated, PD-L1 TPS ≥ 50% NSCLC [5]. Finally, OS rates at 6 months were also similar between the placebo plus pembrolizumab group in this study (81.5%) and the pembrolizumab monotherapy group of the KEYNOTE-024 study (80.2%) [5].

The findings of this study are consistent with results reported in this supplement from the ECHO-306/KEYNOTE-715 study in NSCLC [24] and also with the previously published ECHO-301/KEYNOTE-252 study in metastatic melanoma [22]. ECHO-306/KEYNOTE-715 assessed epacadostat 100 mg BID plus pembrolizumab with chemotherapy in NSCLC [24]; however, enrollment did not require PD-L1 TPS ≥ 50%. The ECHO-301/KEYNOTE-252 study in metastatic melanoma showed that the addition of epacadostat 100 mg BID to pembrolizumab did not improve the primary endpoint of PFS [22].

The pharmacodynamic findings reported here show that circulating kynurenine levels were increased after treatment with pembrolizumab monotherapy. This is consistent with reports suggesting that anti-PD-1 treatment may stimulate IDO1 expression by inducing interferon production [16, 25]. Although epacadostat (≥ 100 mg BID) monotherapy was previously shown to normalize circulating kynurenine levels in patients with solid tumors [19], the addition of epacadostat 100 mg BID to pembrolizumab in our study only reduced pembrolizumab-associated increases in circulating kynurenine levels but did not normalize these levels, and durability of the effect was not evaluated. Similar findings and their interpretation regarding the effects of epacadostat and pembrolizumab on circulating kynurenine levels were reported in patients with urothelial carcinoma [26]. To overcome pembrolizumab-induced kynurenine production, higher doses of epacadostat than those tested in prior monotherapy studies may be needed, and this may be investigated in future combination clinical trials. This rationale is supported by longitudinal plasma kynurenine data from a retrospective pooled analysis of clinical studies evaluating epacadostat in combination with a checkpoint inhibitor. These data showed that epacadostat 100 or 300 mg BID in combination with a checkpoint inhibitor did not control plasma kynurenine levels, whereas epacadostat ≥ 600 mg BID durably controlled plasma and intratumoral kynurenine levels [27]. It should be noted that limitations of plasma kynurenine as a pharmacodynamic biomarker have been described, [28] and studies evaluating other markers to guide epacadostat dose selection may be warranted.

Combined inhibition of IDO1 and programmed cell death protein-1 (PD-1)/PD-L1 were also evaluated in other advanced solid tumors, including the phase I/II ECHO-204 study assessing epacadostat plus PD-1 inhibitor nivolumab [29] and a phase I study assessing IDO1 inhibitor navoximod with PD-L1 inhibitor atezolizumab [30]. These studies have reported preliminary antitumor activity in certain cancers. Clarification of patient populations that could benefit from combined IDO1 and PD-1/PD-L1 inhibition through identification of associated biomarkers could guide further investigation in clinical trials.

This study was limited by its small sample size and short median follow-up. Other limitations include that the study design was changed from phase III to phase II during the study and that the study was discontinued early.

## Conclusions

In this primary analysis, addition of epacadostat 100 mg BID to pembrolizumab therapy for metastatic NSCLC was generally well tolerated but did not demonstrate improved outcomes when compared with placebo plus pembrolizumab. However, the pharmacodynamic findings suggest that further combination studies testing higher doses of epacadostat and using circulating kynurenine levels or other appropriate pharmacodynamic biomarkers to guide dose selection are warranted.

### Supplementary Information

**Additional file1.Supplementary Table 1.** Treatment duration and follow-up.

## Data Availability

Access to individual patient-level data is not available for this study.

## Abbreviations

AE: Adverse event
BICR: Blinded independent central review
BID: Twice daily
C#D#: Cycle # day #
CI: Confidence interval
CR: Complete response
DOR: Duration of response
ECOG PS: Eastern Cooperative Oncology Group performance status
HR: Hazard ratio
IDO1: Indoleamine 2,3-dioxygenase 1
iRECIST: Immune-related Response Evaluation Criteria in Solid Tumors
MedDRA: Medical Dictionary for Regulatory Activities
NCI CTCAE: National Cancer Institute Common Terminology Criteria for Adverse Events
NE: Not evaluable
NR: Not reached
NSCLC: Non-small cell lung cancer
ORR: Objective response rate
OS: Overall survival
PD: Progressive disease
PD-1: Programmed cell death protein-1
PD-L1: Programmed death-ligand 1
PFS: Progression-free survival
PR: Partial response
RECIST v1.1: Response Evaluation Criteria in Solid Tumors version 1.1
SAE: Serious adverse event
SD: Stable disease
TPS: Tumor proportion score
TTR: Time to response
