# Supplementary material for: Epacadostat plus pembrolizumab versus placebo plus pembrolizumab as first-line treatment for metastatic non-small cell lung cancer with high levels of programmed death-ligand 1: a randomized, double-blind phase 2 study
Source: BMC Cancer. 2024 Jul 25;23(Suppl 1):1251. doi: 10.1186/s12885-023-11203-8 (PMC11270761; doi:10.1186/s12885-023-11203-8)
Supplement: Supplementary file 1 — Additional file1.Supplementary Table 1. Treatment duration and follow-up. [file 12885_2023_11203_MOESM1_ESM.docx]

**Supplementary Table 1** Treatment duration and follow-up

|  | Epacadostat + pembrolizumab (*n* = 75) | Placebo + pembrolizumab  (*n* = 77) |
| --- | --- | --- |
| Days on treatment | 172.0 (1.0–350.0) | 168.0 (2.0–362.0) |
| Days on pembrolizumab | 167.0 (1.0–330.0) | 150.0 (1.0–342.0) |
| Days on epacadostat/placebo | 171.0 (1.0–350.0) | 168.0 (2.0–362.0) |
| Cycles of treatment | 8.0 (1.0–16.0) | 8.0 (1.0–17.0) |
| Duration of follow-up, months | 6.8 (0.1–11.4) | 7.0 (0.2–11.9) |
| All data are median (range). | | |
